# Lifestyle behaviours of young adult survivors of childhood cancer

**DOI:** 10.1038/sj.bjc.6600632

**Published:** 2002-11-12

**Authors:** I Larcombe, M Mott, L Hunt

**Affiliations:** Institute of Child Health, UBHT Education Centre, Upper Maudlin Street, Bristol BS2 8AE, UK; Department of Oncology, Bristol Royal Hospital for Children, Upper Maudlin Street, Bristol BS2 8DJ, UK

**Keywords:** childhood cancer, health behaviour, young adults, cancer survivors

## Abstract

This cross-sectional study collected baseline data on the health behaviours of a large population of survivors of childhood cancer in the UK, aged 18–30 years, compared with those of sex- and age-matched controls. Data from 178 young adult survivors of childhood cancer, diagnosed and treated at Bristol Children's Hospital, 184 peers from the survivors' GP practices and 67 siblings were collected by postal questionnaire. Conditional logistic regression analysis showed that, for matched sets of survivors and controls, survivors of a variety of childhood cancers reported lower levels of alcohol consumption (*P*=0.005), lower levels of cigarette smoking (*P*=0.027) and lower levels of recreational drug use (*P*=0.001) than controls. Analysis of matched sets of survivors and siblings showed similar trends but no significant differences. A health behaviour index for each participant was constructed from the data collected on five key health behaviours which influence future health status. Comparison of the means for each case group showed that survivors of childhood cancer were leading healthier lives than controls or siblings. This finding was expressed most clearly as the difference in the means of the health behaviour index for each case group, derived from five health behaviours (one-way ANOVA, *P*<0.001).

*British Journal of Cancer* (2002) **87**, 1204–1209. doi:10.1038/sj.bjc.6600632 www.bjcancer.com

© 2002 Cancer Research UK

## 

It is only in the last 30 years that advances in the treatment of childhood cancer have resulted in the majority of patients surviving into adulthood, with the probability of being able to live a normal life-span. Recent research (Coleman *et al*, 1999, cited in CRC, 1999) shows that several childhood cancers now have survival rates in excess of 75%. However, an increased risk of early death from causes other than the original cancer for survivors, when compared with their siblings, has been found (Nicholson *et al*, 1994). There is also considerable evidence to show that survivors of childhood cancer are at increased risk of developing other late-effects including a second cancer. These may be due to a combination of genetic factors (Li *et al*, 1988) and/or the consequences of damage to various organs caused by the original treatment (Hawkins and Stevens, 1996).

It is well known that lifestyle factors, including smoking, consumption of alcohol, diet, sexual and sun behaviour, influence the risk of developing cancers and other health conditions in the general population. Since survivors of childhood cancer have been shown to be a population susceptible to further ill-health, it is likely that non-avoidance of known ‘risk’ health behaviours by this vulnerable population of young people may further increase this risk. Until recently, little research has been carried out on lifestyles of survivors, but with increasing numbers reaching adulthood their adjustment and assimilation into the community and their health behaviours have become a focus for research. Do the health behaviours and lifestyles of survivors of childhood cancer differ from those of their peers, and if so, how? No British data is available on levels of risk health behaviours of survivors compared with the normal youth population.

American health behaviour research has tended to concentrate on smoking (Corkery *et al*, 1979; Haupt *et al*, 1992; Tao *et al*, 1998). Hollen and Hobbie (1993) and later Mulhern *et al* (1995) did investigate several ‘risk’ health behaviours, but only with small samples in both cases. Mulhern *et al* (1995) found that while 47.5% of survivors had tried smoking, only 17.5% were current smokers. In their later study, Tao *et al* (1998) reported 23% had tried smoking and 19% were current smokers. Figures for the consumption of alcohol by survivors of childhood cancer, aged 12–19 years (Hollen and Hobbie, 1993) were almost half that of school students of comparable age (25 and 45% respectively) and Gray *et al* (1992) found that survivors were less likely than their peers to be drinkers. In their study, Mulhern *et al* (1995) found that, although nearly 75% of their sample had reported some alcohol use, the incidence of ‘problem’ drinking was low. The only data on use of illicit drugs by survivors of childhood cancer (Hollen and Hobbie, 1993) found that 17% of survivors aged 12–19 years had tried marijuana, but none were current users at the time of the survey.

In their study of 40 young adult survivors of childhood cancer, aged 18–29 years, Mulhern *et al* (1995) investigated the health beliefs and behaviours of survivors, and the relationships with various demographic variables. The levels of risk taking behaviours, as evidenced by smoking and drinking, were found to be low and did not appear to be correlated with socio-demographic variables. Together with other health behaviours, diet, sleep, exercise and tooth brushing, also investigated by Mulhern *et al* (1995), the findings showed that young adult survivors have lifestyles that are at least as healthy as, if not more healthy than, young persons of similar age in the general population. That study was the first to address these important issues in young adult survivors.

This study reports the results of a postal survey of health behaviours in a population of young adult survivors of childhood cancer, treated at one specialist centre in the UK, compared with matched controls and a group of siblings. While the study investigated several health behaviours, this study concentrates on the levels of addictive behaviours, cigarette smoking, consumption of alcohol and use of recreational drugs. Data on a total of five health behaviours were analysed to calculate a health behaviour index for comparisons between the three groups.

## MATERIALS AND METHODS

The participants consisted of a population of young adult survivors of childhood cancer, treated at the Bristol Royal Hospital for Sick Children (BCH) and listed on the Bristol cancer registry. Survivors were alive and well, aged 18–30 years and diagnosed at least 5 years before the start of the study in 1996.

A new A5 questionnaire booklet, the *Healthy Living Questionnaire*, was developed specifically for the study. It concentrated on ‘risk’ health behaviours defined in this study as any health behaviour which increases the chances of a person developing health problems as a result of that behaviour. The ‘risk’ health behaviours reported here are drinking alcoholic drinks, smoking cigarettes, using recreational drugs, eating an unhealthy diet and inadequate skin protection. For each behaviour, questions investigated past and current practice and current levels of daily and weekly consumption. For sun behaviour, respondents were asked how many times they were sunburnt in the last 12 months. A separate section investigated self-esteem using the Robson Self-Esteem Questionnaire (Robson, 1989) and locus of control using the Multidimensional Health Locus of Control Scale (Wallston *et al*, 1978).

The new questionnaire was pilot tested with a group of young adult survivors in another region of England and subsequently letters of invitation to take part in the research study and questionnaire booklets were sent to 296 survivors in SW England whose addresses could be traced. After two reminders were sent, 1 month and 2 months after the initial mailing, and a final telephone reminder, 4 months after the initial mailing, 178 completed questionnaires were received. There were 63 non-replies, 39 who refused consent and 16 who did not return the booklet after having given consent, resulting in a response rate of 60%. Since such a loss of the potential population could have caused a bias in the end sample, comparisons between the responder and non-responder populations were conducted on known data about them.

One hundred and eighty-four controls, matched by sex and age (±10 months) were recruited from the same general practices as each of the matched survivors. Response varied between practices and overall 23% of those invited took part. Survivors were invited to recruit their own siblings but the potential sibling number was not known. Sixty-seven siblings of consenting survivors, within the same age range, 18–30 years, but not necessarily of the same sex, also took part in the study.

‘Current drinking’ was defined as drinking alcohol more frequently than only on special occasions. Consumption of alcohol was calculated as ‘units’ of alcohol. ‘Binge’ drinking was defined as 6 or more units of alcohol on any day in the last month. ‘Current smoking’ was defined as smoking at least one cigarette per week. ‘Current recreational drug use’ was defined as using recreational drugs currently, even if this was less often than once a month. Users of recreational drugs were asked to name any drugs used and these were coded as Class A, B or C, according to how harmful they were, using a list supplied by TACADE (1996). Class A drugs, the most harmful, included heroin, cocaine and LSD, Class B drugs included amphetamines and cannabis and Class C drugs included tranquillisers.

Preliminary statistical comparisons between two groups included χ^2^ tests (using a continuity correction for 2×2 tables or Fisher's Exact test if numbers were small) and, for continuous variables, the non-parametric Mann-Whitney *U*-tests. Further comparisons between the survivors and controls, and survivors and siblings, were carried out using a series of univariate Conditional Logistic Regression analyses. These effectively made comparisons within the matched sets of data derived from one survivor and at least one control. The statistical significance was assessed using the ‘Likelihood-ratio test’. The number of survivors with one control was 50 and the number with two or more controls was 63, i.e. there were 113 matched sets. In the equivalent analysis comparing survivors and siblings, the number of survivors with at least one sibling was 49 and the number with two or more siblings was eight, giving 57 matched sets. The number of matched sets in this latter analysis was therefore relatively small, yielding a lower power to detect any differences between these two groups.

### Health behaviour index

A health behaviour index (HBI) was developed in an attempt to calculate the degree to which any individual reported practising overall ‘healthy’ or ‘unhealthy’ behaviour. Six health behaviours known to influence future health status, namely smoking, drinking alcohol, recreational drug use, diet, exercise and sun care, were selected to construct an HBI. The answers to the behaviour questions were arbitrarily graded on a four-point scale from the ‘most healthy’ to the ‘least healthy’ as shown in Table 1

**Table 1:**
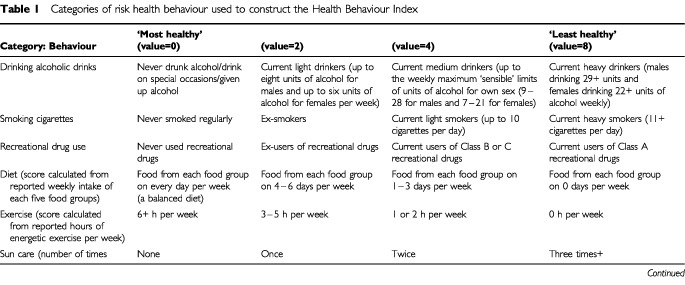
Categories of risk health behaviour used to construct the Health Behaviour Index

. Initially the HBI score for each individual was calculated simply by adding the grades for the six items. A low score indicated overall ‘healthy’ behaviour and a high score indicated overall ‘unhealthy’ behaviour.

In order to refine the HBI values, a combination of optimal scaling and principal components analysis (Young *et al*, 1978) as implemented in SAS User's Guide (Sas Institute Inc., 1989) was carried out. We used the first principal component score to best represent the set of health behaviours. In this analysis, exercise was not found to contribute to the first principal component and so the analysis was repeated using the five remaining variables. Data from all cases (survivors, controls and siblings) were combined for the analysis since separate analyses for these groups had been found to give similar results.

The ‘optimal scaling’ part of the analysis effectively replaced the arbitrarily assigned scores in Table 1 (i.e. the values 0, 2, 4, 8) by scores which better represented each category's importance. A monotonic scaling was used. The ‘principal component’ part of the analysis appropriately weighted the contribution of each of the five health behaviours to yield a new HBI score. (Full details are given in Larcombe, 2001). The first principal component accounted for 37% of the variance in the data.

The mean HBI scores for the three groups were calculated and compared using one-way Analysis of Variance (ANOVA), followed by pair-wise comparisons using Scheffés test. Student's *t*-tests and correlation coefficients were used to explore other factors related to the HBI, and linear regression analysis was used to confirm their independent contribution.

## RESULTS

### Responders* vs* non-responders

Forty-six per cent of traceable male survivors were non-responders compared with 32% of traceable female survivors (*P*=0.022). A similar bias was found in the recruitment of controls (*P*<0.001). Response rates for survivors did not differ significantly between seven major cancer groups (data not shown, *P*=0.877). Survivors of CNS tumours were under-represented in the study population since the majority of cases were treated at another centre. The mean age of responders at diagnosis was 8.2 years (range 0–17) compared with 7.2 years (range 0–19) for non-responders (*P*=0.05) and the mean age of responders at survey was 25.2 years compared with 24.1 years for non-responders (range for both groups 19–32 years; *P*=0.01) but these differences were not thought to be important in influencing consent to taking part in the study.

The mean age of the siblings was 26 years compared with 25 years for the survivor and control groups.

### Drinking, smoking and drug use

Fewer survivors than controls reported being current drinkers (Table 2

**Table 2:**
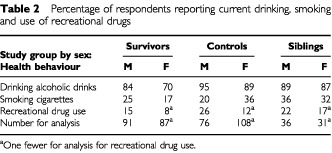
Percentage of respondents reporting current drinking, smoking and use of recreational drugs

; *P*=0.042 for males and *P*=0.002 females respectively). Although fewer survivors than siblings reported being current drinkers this difference did not achieve significance (*P*=0.625 and *P*=0.104 for males and females). Fewer male survivors than their same sex siblings were current smokers but this difference was not statistically significant (*P*=0.316). Amongst females, fewer survivors were current smokers than either their controls (*P*=0.006) or female siblings (*P*=0.133). Fewer survivors than controls or siblings reported being current drug users but none of these results were significant.

Overall, fewer survivors were found to be ‘binge’ drinkers (37%) than controls (42%) or siblings (43%). Binge drinking was higher in males than in females in each group, but none of the differences were significant (data not shown). When looking at consumption of units of alcohol per week, more male survivors (43%) than controls (33%) or siblings (25%) reported light drinking, up to 8 units per week. Similarly in females, more survivors (68%) than controls (53%) or siblings (45%) reported light drinking, up to 6 units of alcohol per week.

### Comparison of matched sets

Both sexes were combined in this series of conditional logistic regression analyses. In the comparison between survivors and their sex/age-matched controls, a significant difference was found with respect to drinking status (Table 3

**Table 3:**
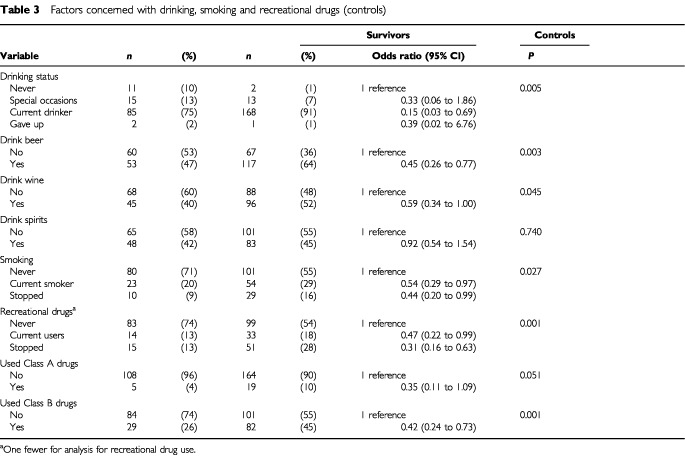
Factors concerned with drinking, smoking and recreational drugs (controls)

). From the odd-ratios shown, survivors were less likely ever to have had a drink than the controls. In particular, they were less likely to be current drinkers. Survivors were less likely than controls to drink beer or wine but there were no differences between the groups for drinking spirits. Survivors were significantly less likely than controls to have ever smoked cigarettes or to be current smokers. However, fewer survivors than controls had managed to stop smoking. Survivors had a lower overall consumption of cigarettes than controls (*P*=0.024, data not shown). Significant differences were found with respect to the use of recreational drugs; survivors were less likely than controls to have ever used recreational drugs and were also less likely to be current users. Survivors were less likely than controls to be using the most risky Class A drugs; the difference was only marginally significant but the numbers were small. Survivors were also less likely to be using Class B drugs, which were almost exclusively cannabis, but here the difference was highly significant. There were very few users of Class C drugs in any of the groups and the differences were not significant (data not shown).

Results for matched sets of survivors and siblings are not reported here since the differences were not statistically significant. The general picture, however, showed that survivors were less likely than siblings to drink alcoholic drinks, to be smokers or ex-smokers, or to have used recreational drugs.

### Health behaviour index

The initial HBI score (based on the arbitrary variable weightings shown in Table 1) and a refined HBI score (using the optimal scaling/principal component analysis) gave similar results; only the latter results are shown here.

The mean (s.d.) HBI scores for the three groups were as follows: survivors −0.31 (s.d. 1.31), controls 0.21 (s.d. 1.34) and siblings 0.25 (s.d. 1.42). There were significant differences between the 3 groups overall (*P*<0.001); Scheffé's tests (at the 5% level) showed significant differences between the means for survivors and each of the other two groups, but not for the controls *vs* the siblings.

A number of variables were found to be related to the HBI index and these included sex, age and marital status. Males had a significantly higher mean score (*P*=0.001) and there was a weak negative correlation with age (*r*=−0.114, *P*=0.019). The mean score, furthermore, was significantly higher if the respondent was single rather than married/divorced or separated (*P*=0.001). The difference was found to be independent of age and sex. The HBI was also correlated with indices than measured ‘self-esteem’ and ‘locus of control’ (details not shown here but see Larcombe, 2001). More of the survivors were single (68% compared with 52% of the controls and 58% of the siblings) and in general they were found to have lower self-esteem and lower internal locus of control. The Conditional Logistic Regression analyses were repeated using HBI, adjusting for marital status, self-esteem and locus of control; there were significant differences in HBI scores between survivors and controls (*P*=0.002) and between survivors and siblings (*P*=0.040).

These results indicate that survivors have reported, and thus may be living, a healthier lifestyle than either controls or siblings when the five health behaviours included in the HBI are taken into account.

## DISCUSSION

The focus of this cross-sectional study was to collect baseline data on the lifestyle health behaviours of young adult survivors of childhood cancer. The objectives were to compare the levels of risk health behaviours of survivors with those of sex- and age-matched controls and a group of siblings. The response rate of 60% from traceable survivors compares with 57% obtained by Mulhern *et al* (1995) in their postal survey.

Comparisons of sex, cancer type and age at diagnosis, which were available from the hospital notes, and age at time of study, showed no significant differences between the responders and non-responders. This evidence, and that obtained by Bynner *et al* (1997), suggest that populations of responders and non-responders essentially do not differ. Survivors of CNS tumours were under-represented in the current study, because the majority of cases were not treated at the paediatric regional centre. The same reason for non-inclusion of CNS survivors was also reported by Green *et al* (1991).

The main shortcoming in recruitment for this study was a lower response rate for males compared with females in both the survivor and control groups. Similar sex differences in response rates, where females have been more helpful than males, have been recorded in other youth studies (Prescott-Clarke and Primatesta, 1998; Bynner *et al*, 1997). Research studies of risk health behaviours have shown that higher proportions of males than females exhibit risk behaviours (Ford *et al*, 1997; Prescott-Clarke and Primatesta, 1998). To the extent that this represents a significant selection bias, the levels of adoption of unhealthy behaviours reported in this study may be underestimated. The total number of siblings recruited was low which decreased the power of the comparison tests and gave less confidence in interpreting significance tests between the survivor and sibling groups.

Inter-group comparisons showed that fewer survivors were current drinkers than controls or siblings, and comparisons of matched sets confirmed a significantly lower alcohol consumption for survivors compared with controls. These results support the findings from the Canadian and American studies of survivors (Gray *et al*, 1992; Mulhern *et al*, 1995). Fewer survivors than controls or siblings regularly exceeded the limit of 5 units of alcohol at a single sitting (binge drinking), and it appeared that overall, survivors had lower weekly alcohol consumption than controls or siblings. In addition to those survivors who reported never having drunk alcohol, others reported that they drank alcoholic drinks only on special occasions. These findings help explain the lower consumption of alcohol found in the survivor group compared with controls or siblings. It would appear that survivors, as a group, are more moderate drinkers than their peers.

Inter-group comparisons of smoking behaviour and comparison of the matched sets showed that survivors were significantly less likely than controls to have ever smoked cigarettes or to be current smokers. These results are comparable with the findings reported by Mulhern *et al* (1995) and Tao *et al* (1998). Of the current smokers, controls appeared to smoke the most heavily, but of the ex-smokers, controls had also been the most successful in stopping smoking. This finding is consistent with Tao *et al* (1998) where smoking survivors were found to be less likely to quit than siblings.

Early American studies of smoking rates in young adult survivors showed that smoking rates were comparable with age-matched population norms (Corkery *et al*, 1979 and Troyer and Holmes, 1988). While smoking rates of young adults have dropped over the last 30 years (Opcs, 1996), along with more recent survivor studies (Mulhern *et al*, 1995; Tao *et al*, 1998), this study has found that rates of smoking for survivors have dropped faster than for comparable age groups in the general population. Comparisons of matched pairs in this study found that 20% of survivors, 29% of controls and 34% of siblings were current smokers compared with 36% of the general population aged 25–31 years (Cox *et al*, 1993). This evidence suggests that survivors, as a group, are choosing not to smoke cigarettes.

Inter-group comparisons showed that significantly fewer survivors of either sex, than controls, had ever used recreational drugs or were current users. Cannabis was found to be the most commonly taken recreational drug, also reported by Ford *et al* (1997), and the proportion of survivors who had used cannabis was comparable with figures reported by Wasserman *et al* (1987). Comparison of matched sets confirmed that only a minority of each case group were ‘current users’ (13% of survivors and 18% of controls; 16% of survivors and 20% of siblings). There are no data from other survivor studies for comparison. However, these figures were lower than the 24% of 16–24 year olds in the general youth population reported by Ford *et al* (1997) as using recreational drugs ‘in the last month’. This evidence suggests that survivors, as a group, are less likely than their peers to experiment with recreational drugs, even with cannabis, the most commonly used.

There have been various attempts by researchers to develop a health behaviour index of health behaviours but so far there has not been a standard questionnaire with which to measure it. Belloc and Breslow (1972) were the first researchers to investigate and define a number of everyday health practices which appeared to affect health and longevity. These health habits included never drinking to excess, never having smoked, weight control, getting adequate sleep, getting adequate exercise, avoiding snacks and eating breakfast. The researchers gave a score of 1 to each of these seven health practices, thus producing a simple scale for measuring healthfulness, from 0 to 7. Although this method of additive scoring of health practices has been widely used (see references in Slater and Linder, 1988), these authors maintained that the approach lacked validity, since much information was lost in collapsing the data and in the scoring process.

This study looked at five major risk health behaviours: smoking, drinking alcohol, use of recreational drugs, unhealthy diet and lack of preventive sun care, and used all the data to score in four categories for each behaviour. In this way, data was not lost and assessment was based on behaviours most likely to present a future risk of ill-health. This study found that survivors as a group had a statistically lower mean for the derived first principle component of the HBI, an indication of healthier behaviour, than either controls or siblings. This finding, that survivors practice healthier behaviours than their peers, is commensurate with the results of the American study of survivors of childhood cancer (Mulhern *et al*, 1995). That females generally behave more healthily than males is a finding which is consistent with the literature (Steptoe *et al*, 1994; Uitenbroek *et al*, 1996; Pavis *et al*, 1998). The finding that increasing age was associated with healthier behaviour supports the consensus of results from other studies (Cox *et al*, 1993; Hill *et al*, 1993; Kinne *et al*, 1996; Malbon *et al*, 1996).

The contribution of this research to the existing literature is in providing further evidence that long-term survivors of childhood cancer are leading healthier lifestyles, in terms of practising more preventive health behaviours, than other young people in the general population. The study has produced baseline data for the UK, on a variety of risk health behaviours of young adult survivors of childhood cancer, with which the results of future studies can be compared. It has been suggested that the differences in rates of drinking, smoking and drug taking found in survivors may be associated with a less active social life. Further research is required to investigate this hypothesis.

This study has four significant limitations. First, with only 48% of the eligible population having completed survey questionnaires, this presents a major problem in terms of self-selection, a process which in general favours females (Bynner *et al*, 1997; Prescott-Clarke and Primatesta, 1998). Self-selection may also have negatively influenced the response of individuals who believed they were leading unhealthy lifestyles (Pietila *et al*, 1995). However, statistical tests showed no significant differences in demographic or disease-related variables, between the responders and non-responders, except for a lesser response by male compared with female survivors and this pattern was repeated in the recruitment of matched controls. The literature reflects similar problems for studies which depend on voluntary participation. Unfortunately, ethical considerations did not allow non-responders to be followed-up in this study.

Second, the results reported in this study excluded survivors of brain and CNS tumours, a group well documented as having special problems (Byrne *et al*, 1989; Lannering *et al*, 1990; Mostow *et al*, 1991; Donahue, 1992). Further research is needed to establish whether the health behaviours of survivors of brain and CNS tumours are comparable with those of other survivors, or whether there are also significant differences in the area of health behaviour.

Third, the research instrument used in the current study was not independently validated. However, the results from the pilot and main studies were comparable (Larcombe, 2001) and they were also comparable with results obtained from young adult American survivors of childhood cancer (Mulhern *et al*, 1995). Although these findings suggest reliability and validity of the ‘*Healthy Living Questionnaire*’, they are limited to the population of young adult survivors treated at the Bristol Children's Hospital. However, since this population can be considered to be representative of survivors treated at other UK Children's Cancer Study Group centres, the results are generalisable to survivors of childhood cancer, excluding brain and other CNS tumours, in the UK.

The fourth limitation concerns the development of the HBI to compare the overall health behaviour between the three case groups. The five health behaviours included in the construction of the HBI were chosen because they pose a risk to future health status. It is acknowledged that the HBI is dependent on the health behaviours included and that a different mix of health behaviours might have resulted in a different pattern of spread in individual HBI values.

Further research is needed to investigate whether survivors choose to practice higher levels of preventive health care than controls, as a positive desire to live a healthy life or as a response to fearing the negative effects of practising unhealthy behaviours. Survivors of brain and CNS tumours form 21% of the population of survivors of childhood cancers (Stiller, personal communication). Research is required to determine their levels of drinking, smoking and other risk health behaviours compared with matched controls.

There is a need for a standard measure of health behaviour which is reliable and valid. Until now, researchers have investigated different numbers of areas of health behaviour, and different levels of each behaviour and used different scoring methods. These measures are not comparable with each other. One of the first decisions to be made should be the selection of health behaviours which should be included in this new measure. A major exercise would be the determination of the number and ranges of categories within each behaviour. Other important considerations would be the appropriate weightings to be given to categories within each health behaviour, and then to each health behaviour compared with the others. Developing a standard instrument for measuring health behaviour, which is quick and easy to use, is much needed in the field of health research.
